# Informing low-risk drinking guidelines: a secondary, cross-sectional data analysis of alcohol use and health utility

**DOI:** 10.1186/s13722-026-00679-x

**Published:** 2026-05-29

**Authors:** Jeremy W. Bray, Arnie P. Aldridge, Abraham Gebreselassie

**Affiliations:** 1 UNC Greensboro, Bryan School of Business and Economics, 449 Bryan Building, PO Box 26170, Greensboro, NC 27402-6170 USA; 2 RTI International, 3040 Cornwallis Road, Research Triangle Park, NC 27709-2194 USA

**Keywords:** Low-risk drinking guidelines, Alcohol consumption, Health utility

## Abstract

**Background:**

Low-risk drinking guidelines seldom incorporate quality of life (QOL). This secondary data analysis explores the relationship between drinking patterns and QoL.

**Methods:**

We estimate the relationship between specific drinking behaviors and QoL, as measured by health utility, which reflects individuals’ preferences for living in states of health. We use cross-sectional data from the US nationally representative National Epidemiologic Survey on Alcohol and Related Conditions-III dataset. We modeled individuals’ health utility as quadratic functions of the typical frequency of drinking and either the typical quantity consumed or the maximum quantity consumed on a single occasion in the past year. All analyses were limited to the target population for most low-risk drinking guidelines—current drinkers with no history of alcohol use disorder. All analyses were stratified by biological sex.

**Results:**

For females, health utility is concave in the frequency of consumption and decreasing in quantity. For males, the relationship is flatter, possibly increasing in frequency, and decreasing in quantity. Results suggest that the greatest health utility is associated with no more than 1 drink per day for women and no more than 3 drinks per day for men, and that, for both sexes, the greatest health utility is associated with drinking on fewer than 7 days per week.

**Conclusions:**

When the health utility of drinkers is considered, the optimal level of drinking is the same or less than many low-risk drinking guidelines. Although descriptive, our results suggest that accounting for drinkers’ health preferences will reinforce current recommendations.

**Supplementary Information:**

The online version contains supplementary material available at 10.1186/s13722-026-00679-x.

## Background

Formal recommendations on alcohol use, called low-risk drinking guidelines, are a staple of alcohol public health efforts worldwide. Health agencies in the US, UK, Canada, Australia, and many other nations have issued low-risk drinking guidelines based on the health effects of consumption [1–3]. Although the scientific basis for guidelines varies by country, all rely on epidemiological evidence of the harmful health effects of drinking to establish limits based on either the absolute or relative risk of mortality or morbidity caused by alcohol use. Because both the relative and absolute risk of harm associated with drinking may vary based on the pattern of consumption, some guidelines specify both daily and weekly limits, although the usefulness of recommending both is a source of debate [4–7]. Many guidelines also recommend populations that should not drink at all, typically children and adolescents, people who are or may become pregnant, and those with an alcohol use disorder [8–10].

Despite the ubiquity of low-risk drinking guidelines, there is little evidence that they impact drinking behaviors. Early research on guidelines focused on public awareness of the guidelines themselves [11, 12]. Later studies assessed the effectiveness of guidelines but failed to find evidence that they reduced consumption [13]. Thus, the current evidence suggests that even when the public is aware of guidelines, they seldom reduce alcohol consumption at a population level. Nonetheless, guidelines still play an important role in alcohol public health messaging. Given their importance in setting consumption targets for alcohol policy and alcohol public health efforts, it is critical to develop low-risk drinking guidelines that are more effective in changing drinking behaviors.

The process for developing low-risk drinking guidelines has been criticized, however, as lacking transparency, being dependent on subjective assessments of health risks that people will tolerate, or as paternalistic attempts to dictate the health risks that drinkers’ should accept [14, 15]. The morbidity and mortality focus of most guidelines ultimately result in guidelines that ignore, or at best indirectly incorporate, drinkers’ quality of life and preferences for health. Research consistently finds that low levels of alcohol use are associated with higher quality of life for the drinker [16, 17], yet no existing guidelines formally incorporate drinkers’ quality of life. Debates about revised guidelines in the US [18] and in Canada [19–22] underscore the continuing tension between drinking limits that focus on increasing years lived rather than on the quality of life in those years or, at a more fundamental level, individuals’ subjective preference for health over other aspects of drinking behaviors, such as social interactions.

Our study helps to address these criticisms by empirically estimating the relationship between specific drinking behaviors and health utility, which is a measure of individuals’ preferences for health-related quality of life. We simultaneously measure both health-related quality of life and individuals’ preferences for health using the SF-6Dv1 measure of health utility. Health utility is an economic construct that reflects individuals’ preferences for living in various states of health. In the case of the SF-6Dv1, those states are defined by the SF-12v2 quality of life instrument. Health utility is defined on a 0–1 scale where 0 reflects the utility of being dead and 1 reflects the utility of ideal health. As the basis for quality-adjusted life years [23], health utility is a well-established measure of individuals’ preferences for health and is therefore an ideal measure to incorporate health-related quality of life and drinkers’ preferences for health into low-risk drinking guidelines. Using data from the National Epidemiologic Survey of Alcohol and Related Conditions-III (NESARC-III), we modeled individuals’ health utility as a quadratic function of the frequency and quantity of alcohol consumed to identify the pattern of drinking that is associated with the highest level of health utility. Our results can be used as an adjunct to epidemiological evidence on the relative and absolute risk posed by differing consumption patterns when setting drinking guidelines, potentially addressing concerns of paternalism in the guideline process.

## Methods

### Data

We conducted a secondary analysis of previously collected cross-sectional data. Data are from the NESARC-III, a nationally representative, cross-sectional survey that was sponsored, designed, and directed by the National Institute on Alcohol Abuse and Alcoholism. When weighted, NESARC-III data are representative of the civilian, noninstitutionalized U.S. population, aged 18 or older, including persons living in noninstitutional group quarters [24]. The NESARC-III used multi-stage probability sampling [24]. In general, the primary sampling unit (PSU) was defined as counties in the US, but some less populous counties were combined to form a PSU. From an initial sampling frame of 2,349 PSUs, 150 were selected using stratified proportional-to-size sampling. Secondary sampling units were defined as area segments based on census blocks or groups of census blocks. 7,200 out of 418,239 segments were sampled, 71,052 addresses/dwelling units were sampled within these segments, and individual persons sampled within these addresses/dwelling units. Data were collected from a final sample of 36,309 adults beginning in April 2012 and ending in June 2013. See Grant et al. [24] for full details on the NESARC-III survey design and data collection.

For this analysis, we limited our analysis to individuals who were classified as current drinkers and had no indication of current or prior alcohol use disorder (AUD) in an attempt to exclude populations that many low-risk drinking guidelines suggest should not drink at all [8–10]. After applying these population inclusion criteria, we had an initial sample of 17,006, consisting of 9,897 females and 7,109 males. After deletion for missing values on alcohol consumption variables (no other analysis variables had missing values), our final analysis sample size is 16,989 (99.9% of the target population sample), consisting of 9,888 females and 7,101 males. Given the low prevalence of missing data in our target population sample, no adjustments were made for missing data. The secondary use of the NESARC-III data was initially approved by the UNCG IRB on November 13, 2018 (study # 19–0237).

#### Health utility

Health utility is measured with the SF-6Dv1 as derived from selected items from the SF-12v2 [25]. The SF-6Dv1 is a community preference weighted measure of health utility [25–27]. The psychometric properties of the SF-6Dv1 have been tested in numerous disease areas [28]. Importantly, research suggests that the SF-6Dv1 is particularly sensitive to mild health conditions [28–30], whereas other utility measures are not, making it particularly suitable to use for the full spectrum of alcohol consumption states. Furthermore, the primary alternative to the SF-6D, the EQ-5D, does not perform well for some alcohol health states [31]. We use a UK weighting function for the SF-6Dv1 [25]. Although US population weights for the SF-6Dv1 have been derived based on the SF-36 [27], they cannot be applied to the SF-12v2 and so are not used in this analysis. No US weighting function exists to calculate any version of the SF-6D from the SF-12v2 and so the UK weighting function was the only one available. For our analysis, we multiplied SF-6Dv1 scores by 100 to improve the scaling and interpretability of our results.

The SF-6Dv1 has at least two advantages as a measure of health-related quality of life in the context of our analyses. First, it is a single measure of health-related quality of life. The underlying SF-12v2 from which we calculate the SF-6Dv1 does not have a single summary measure but rather has two summary scores: the PCS for physical health conditions and the MCS for mental health conditions [32]. Second, the SF-6Dv1 incorporates population-level preference weights so that it provides information that is, arguably, more relevant to the drinking decision of individuals. That is, it allows us to tell consumers how drinking patterns relate to a quality of life that they would actually prefer over another, and not just how their drinking relates to an abstract quality of life score.

#### Alcohol consumption

Alcohol consumption is measured using a series of questions on drinking in the past year that capture four primary drinking behaviors: typical quantity consumed when drinking, frequency of consuming the typical amount, maximum quantity consumed on any one occasion, and the frequency of heavy episodic drinking, i.e., binge drinking. The NESARC-III first assesses typical frequency by asking respondents about how often they drank any kind of alcoholic beverage in the past 12 months. Respondents were given 10 categorical response options ranging from “every day” to “1 or 2 times in the past year.” Using the midpoints of these categories, we created a continuous measure of typical frequency in the past year, which we then divided by 52 to create a weekly measure of typical frequency. NESARC-III also asked respondents how many drinks they usually had on days when they drank as a continuous response question, which we used as our measure of typical quantity. To help respondents estimate their usual drink size, the NESARC-III used flashcards that included life-sized photographs of common glasses for a variety of alcohol types [33]. Respondents were then asked what was the largest number of drinks they consumed on a single day. Respondents again provided that number as a continuous response, which we used as our measure of maximum quantity. Because we limit our analysis sample to respondents that the NESARC-III classifies as current drinkers, variables measuring typical frequency, typical quantity, and largest quantity are strictly positive (i.e., they do not include zeros). Finally, respondents were asked how often they drank 4 or more drinks in 2 h or less in the past year and how often they drank 5 or more drinks in 2 h or less in the past year, with the same response options as for typical frequency. Using these variables, we created a categorical variable for the frequency of binge drinking, including a category for no binge drinking in the past year, defining binge drinking as 4 or more drinks for females and 5 or drinks for males.

#### Comorbid health conditions

NESARC-III collects data on 46 physical and behavioral health conditions. Physical health conditions in the past 12 months are self-reported by respondents as having been “diagnosed by a doctor or other health professional.” Behavioral health conditions, including alcohol use disorder, are measured using the AUDADIS-V [34] and are assessed as occurring in the past year or prior to the past year, making a lifetime diagnosis possible. Controlling for these conditions may help to disentangle the role of morbidity in health utility from other positive aspects of drinking such as socialization. Appendix A lists these conditions and their prevalence in NESARC-III.

#### Demographics

We include a set of demographic covariates based on prior empirical research on the predictors of health-related quality of life, health utility, or adverse alcohol-related health consequences [35–38]. Relevant demographic variables in the NESARC-III used in our analyses include biological sex, age, race/ethnicity, marital status, and household income. We stratify all analyses by biological sex as recorded in NESARC-III because some drinking guidelines vary by sex [39]. The NESARC-III only asks respondents to self-report their binary sex “…if it’s not obvious to the interviewer” and does not collect data on transgender or hormone replacement therapy. Given the context and time frame in which the data were collected, respondents’ sex reported in the NESARC-III most likely approximates biological sex, but this may not be the case for all respondents. All other variables are included as categorical covariates in all models. Response categories for age, race/ethnicity, and marital status are as provided in the NESARC-III data files. Using the observed distribution across the categorical household income variable included in the NESARC-III, we created indicators for the approximate lower 25% ($14,000 per year and below) and the upper 25% (above $65,000 per year) of the annual household income distribution. The middle 50% of the distribution serves as the reference category.

### Methods

We model individuals’ health utility as a quadratic function of the typical quantity and typical frequency of alcohol consumption. Specifically, we estimate the following regression model using ordinary least squares (OLS):

$$\eqalign{& {{\rm{U}}_{\rm{i}}}{\rm{ = }}{{\rm{\beta }}_{\rm{0}}}{\rm{ + }}{{\rm{\beta }}_{\rm{1}}}{\rm{T}}{{\rm{Q}}_{\rm{i}}}{\rm{ + }}{{\rm{\beta }}_{\rm{2}}}{\rm{TQ}}_{\rm{i}}^{\rm{2}} \cr & \,\,\,\,\,\,\,\,{\rm{ + }}{{\rm{\beta }}_{\rm{3}}}{\rm{T}}{{\rm{F}}_{\rm{i}}}{\rm{ + }}{{\rm{\beta }}_{\rm{4}}}{\rm{TF}}_{\rm{i}}^{\rm{2}}{\rm{ + }}{{\rm{\beta }}_{\rm{5}}}{\rm{T}}{{\rm{Q}}_{\rm{i}}} \cr & \,\,\,\,\,\,\,\,\,\,\,{\rm{ \times T}}{{\rm{F}}_{\rm{i}}}{\rm{ + }}{{\rm{\beta }}_{\rm{6}}}{{\rm{X}}_{\rm{i}}}{\rm{ + }}{{\rm{\varepsilon }}_{\rm{i}}} \cr} $$

where U_i_ is the SF-6Dv1 health utility for individual i, TQ_i_ is typical quantity of alcohol consumed and TF_i_ is the typical frequency of consuming alcohol, X_i_ is a vector of control variables, ε_i_ is the regression error term, and the βs are coefficients to be estimated. Because the square of a variable is highly colinear with its linear term, we present tests of joint significance for the coefficients on each linear/square combination.

In addition to this specification, we estimate models that control for the frequency of binge drinking as a categorical variable, and models that use a polynomial in typical frequency and largest quantity. All models are estimated both with and without controls for the 46 confounding health conditions assessed in the NESARC-III. All models are estimated using survey commands in Stata version18 and so use sampling weights and account for the multi-stage sampling design of the NESARC-III.

We chose a quadratic function given its ease of implementation and interpretation. Any given functional form is as restrictive as any other, but the quadratic function can be viewed as a second order Taylor series approximation of any, unknown functional form and so is an appropriate choice for our descriptive analyses. Nonetheless, it may force the nonlinear relationship to turn up or down, rather than asymptote. If this happens over the logical range of consumption (e.g., 0 to 7 days per week), then the quadratic form may yield biased conclusions about the association between drinking behaviors and health utility. To explore this possibility, we calculate the inflection points of the function to understand over what range of alcohol consumption the quadratic function may be forcing a change in the general direction of the relationship. The overall patterns are characterized in surface plots showing the relationship between health utility and consumption behaviors over the logical ranges of quantity and frequency.

### Sensitivity analyses

We conducted sensitivity analyses to explore three key issues that may impact our results. First, as previously mentioned, there is no US utility weighting scheme that supports calculation of any version of the SF-6D from the SF-12v2. As a result, we used UK preference weights that may not reflect the preferences of the US population. To further explore this concern, we also estimated our regression models using the SF-12v2 PCS and MCS as outcome variables. Second, the complex trajectories of drinking over the life course may impact the estimated relationship between alcohol use and health utility, especially since people tend to reduce their alcohol consumption as they become unwell or frail with advancing age. To explore this concern, we estimated models stratified by three age categories, each with approximately one third of our analysis sample: 18 to 35, 35 to 50, and 51 and older. Third, many policymakers and researchers are exploring non-abstinence targets for alcohol use disorder treatments such as pharmacotherapies [40, 41]. To explore the connection between health utility and drinking patterns for individuals with alcohol use disorder, we estimated models using the subpopulation of individuals who the NESARC-III classifies as having an alcohol use disorder, either in the past year or prior to the past year, based on the AUDADIS-5.

## Results

Table 1 presents descriptive statistics for key variables used in our analysis. The average health utility is 78.994 among biological females and 82.266 among biological males. On average, biological females drink on just over 1 day per week and consume just under 2 drinks per occasion. Biological males consume alcohol a little less than 2 days per week and usually drink about 2.5 drinks per occasion. Both biological females and males are approximately evenly distributed over the age categories, although both have a slightly lower proportion in the 51- to 60-year-old age range. As expected for a US sample, the majority of both biological females and males are White, non-Hispanic. Just over half of both samples are married or living with someone as if married.

**Table 1:** Sample descriptive statistics for NESARC-III respondents with no lifetime AUD and who consumed at least 1 drink in the past 12 months

	Biological females	Biological males
*n*	9,888	7,101
SF-6Dv1	78.994	82.266
	(0.002)	(0.002)
Typical frequency	1.045	1.606
	(0.024)	(0.030)
Typical quantity	1.830	2.528
	(0.020)	(0.036)
Largest quantity	2.931	4.532
	(0.032)	(0.058)
Age		
18–35	31.8%	31.1%
36–50	27.6%	26.8%
51–60	17.3%	18.4%
61+	23.3%	23.7%
Race/ethnicity		
White, non-Hispanic	67.4%	65.4%
Black, non-Hispanic	11.8%	10.6%
American Indian/Alaska Native, non-Hispanic	0.6%	0.6%
Asian/Native Hawaiian/Other Pacific Islander, non-Hispanic	3.8%	5.8%
Hispanic, any race	16.5%	17.8%
Marital status		
Married	42.7%	48.8%
Living with someone as if married	5.9%	5.8%
Widowed	9.3%	4.1%
Divorced	16.1%	12.1%
Separated	4.4%	3.0%
Never married	21.6%	26.2%
Annual household income		
≤ $14,000	27.2%	22.2%
$14,001 to $65,000	48.9%	48.6%
> $65,000	23.9%	29.2%

Linearized standard errors that account for the NESARC-III multistage sampling design are in parentheses

Table 2 presents regression coefficients for the alcohol consumption variables for biological females. The first two columns present results for our base specification of typical frequency and typical quantity, the third and fourth column present results from models that also control for the frequency of bingeing, and the last two columns present results for models that include the largest quantity rather than the typical quantity. In each pair of columns, the left column omits the comorbid health condition control variables while the right column includes them.

**Table 2:** SF-6Dv1 regression results for biological females as a function of alternative frequency and quantity of consumption measures

	Typical frequency and typical quantity		Typical frequency and typical quantity with bingeing controls		Typical frequency and largest quantity	
	Without health controls	With health controls	Without health controls	With health controls	Without health controls	With health controls
Typical frequency	2.081^***^	0.987^***^	2.252^***^	1.105^***^	1.912^***^	0.785^**^
	(0.313)	(0.259)	(0.328)	(0.274)	(0.314)	(0.268)
Typical frequency^2^	-0.253^***^	-0.113^**^	-0.261^***^	-0.118^**^	-0.250^***^	-0.104^*^
	(0.0476)	(0.0396)	(0.0485)	(0.0401)	(0.0487)	(0.0402)
Typical quantity	-0.399	-0.190	-0.347	-0.141		
	(0.227)	(0.203)	(0.230)	(0.209)		
Typical quantity^2^	-0.0143	-0.0166	-0.0193	-0.0207		
	(0.0220)	(0.0214)	(0.0214)	(0.0208)		
Typical frequency ⋅ typical quantity	-0.0313	-0.0520	-0.119	-0.109		
	(0.0511)	(0.0486)	(0.0617)	(0.0603)		
Largest quantity					-0.162	-0.0510
					(0.0994)	(0.0897)
Largest quantity^2^					-0.00184	-0.00281
					(0.00313)	(0.00291)
Typical frequency ⋅ largest quantity					0.0246	0.0108
					(0.0355)	(0.0334)
**χ**^**2**^ **tests of joint significance**						
Typical frequency and typical frequency^2^	22.96	7.90	24.70	8.79	18.48	4.25
p value	0.0000	0.0006	0.00	0.0003	0.0000	0.0166
Typical quantity and typical quantity^2^	6.14	2.71	5.20	2.32		
p value	0.0029	0.0706	0.0069	0.1029		
Largest quantity and largest quantity^2^					4.61	2.08
p value					0.0119	0.1296
All drinking	13.24	4.97	14.89	5.93	11.42	2.38
p value	0.0000	0.0004	0.0000	0.0001	0.0000	0.0433
**Inflection points**						
Typical frequency	5.33	8.88	21.34	-29.13	2.48	3.65
Typical quantity	-19.77	-19.61	-74.71	73.30		
Largest quantity					-27.29	-2.06

*N* = 9,888 Linearized standard errors that account for the NESARC-III multistage sampling design are in parentheses. All models control for age, race/ethnicity, household income, and marital status^*^p<.1; ^**^p<.05; ^***^p<.01

Across all model specifications, we see a robust and statistically significant relationship between the frequency of drinking and health utility for biological females based on the individual coefficient estimates (*p* <.01 in all models for both frequency and frequency squared). The frequency of drinking has an overall positive association with health utility (e.g., typical frequency = 2.081, *p* <.01) but at a decreasing rate (e.g., frequency squared = − 0.253, *p* < .01). The strength of the relationship between frequency and health utility is further supported by the joint tests of significance (frequency and frequency squared, *p* <.01 in all models). Although the individual coefficients for quantity of drinking are not significant, the joint tests of significance suggest a significant relationship when comorbid health conditions are not controlled for. Across all specifications, controlling for comorbid health conditions attenuates the individual coefficients but does not change their sign or, in most cases, their significance. The implied inflection points for both frequency and quantity of drinking are close to or outside the logical range of alcohol consumption for most specifications — the points must be interpreted as a pair, not individually, given that all models include an interaction between frequency and quantity.

**Fig. 1:**
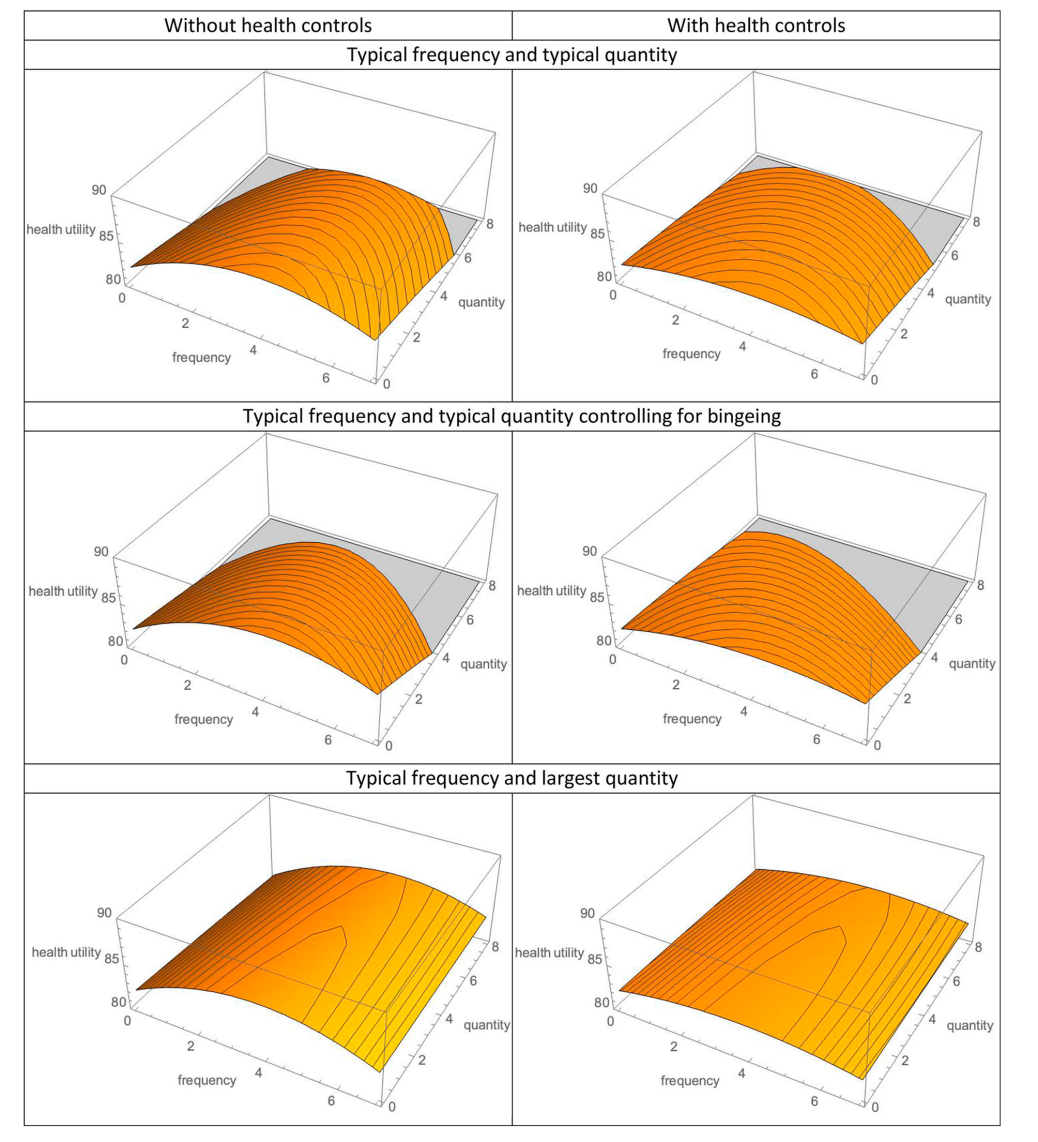
Surface plots of regression results for biological females

To provide a better understanding of the relationships being estimated, Fig. 1 presents surface plots of the relationship between drinking and health utility plotted over a range of 80 to 90 for health utility, 0 to 7 for frequency per week and 0 to 8 for quantity per occasion. The generally accepted minimally important differences in health utility range from 0.03 to 0.07 [42–44], or 3–7 in our surface plots. All plots show a similar shape of an inverted U in frequency that is downward sloping in quantity. Overall, these results suggest that health utility is declining in quantity but has a peak in frequency at between 3 and 6 days per week. The differences in utility over the range of both frequency and quantity are greater than the minimally important threshold of 3 in most surface plots, especially for quantity.

Table 3 presents regression results for biological males. Controlling for comorbid health conditions attenuates the coefficients in all models to the point that none are individually significant at *p* < .10. Tests of joint significance, however, show that the relationship between health utility and frequency of drinking is significant in all but the last two models. Importantly, the sign on the squared frequency term is negative when health controls are not included but positive when they are. The relationship between quantity consumed and health utility is never significant. Although most of the inflection points are out of logical range, the inflection points for models that control for comorbid health conditions are close to or within plausible ranges. Thus, these models may impose binding functional form constraints.

**Fig. 2:**
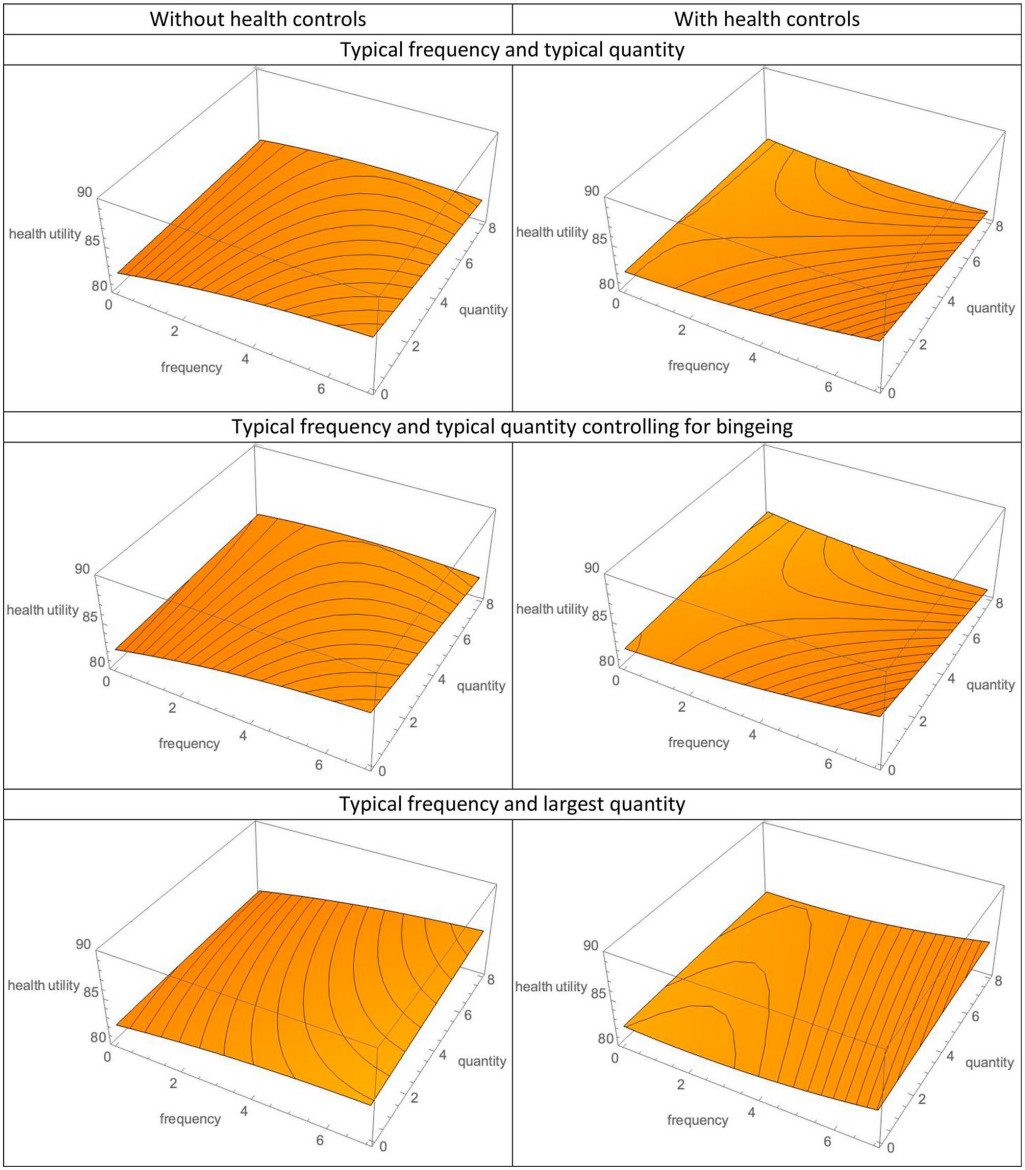
Surface plots of regression results for biological males

Figure 2 presents surface plots for biological males. Figure 2 shows a more complex relationship for biological males than was observed for biological females. The fundamental shape of the relationship is sensitive to the inclusion of controls for comorbid health conditions, highlighting the importance of the sign of the squared frequency term. All models that use the typical quantity consumed suggest that, for biological males, health utility is maximized at 1 drink per day consumed on 5 to 7 days per week. Models that use the largest quantity consumed, however, suggest that health utility is upward sloping in both frequency and quantity. Differences in utility over the ranges of frequency and quantity are not as striking as they were for biological females and may not be meaningful in some models.

**Table 3:** SF-6Dv1 regression results for biological males as a function of alternative frequency and quantity of consumption measures

	Typical frequency and typical quantity		Typical frequency and typical quantity with bingeing controls		Typical frequency and largest quantity	
	Without health controls	With health controls	Without health controls	With health controls	Without health controls	With health controls
Typical frequency	0.780^**^	0.114	0.798^**^	0.162	0.466	-0.177
	(0.293)	(0.273)	(0.299)	(0.279)	(0.300)	(0.285)
Typical frequency^2^	-0.0416	0.0419	-0.0429	0.0377	-0.0317	0.0529
	(0.0452)	(0.0426)	(0.0454)	(0.0431)	(0.0467)	(0.0441)
Typical quantity	0.0125	0.0124	0.0560	0.0818		
	(0.154)	(0.136)	(0.164)	(0.142)		
Typical quantity^2^	0.000723	0.000878	0.000121	-0.000158		
	(0.00474)	(0.00422)	(0.00474)	(0.00417)		
Typical frequency ´ typical quantity	-0.0510	-0.0669	-0.0591	-0.0809		
	(0.0446)	(0.0396)	(0.0481)	(0.0410)		
Largest quantity					0.0769	0.0662
					(0.0991)	(0.0971)
Largest quantity^2^					-0.00471	-0.00288
					(0.00393)	(0.00413)
Typical frequency ´ largest quantity					0.0194	0.00560
					(0.0244)	(0.0203)
c^2^ tests of joint significance						
Typical frequency and typical frequency^2^	10.17	5.85	9.59	6.35	2.33	1.30
p value	0.0001	0.0038	0.0001	0.0024	0.1024	0.2758
Typical quantity and typical quantity^2^	0.06	0.08	0.17	0.33		
p value	0.9423	0.9195	0.8455	0.7176		
Largest quantity and largest quantity^2^					0.71	0.27
p value					0.4930	0.7648
All drinking	7.08	3.07	6.63	2.93	6.43	1.80
p value	0.0000	0.0124	0.0000	0.0162	0.0000	0.1192
Inflection points						
Typical frequency	0.65	0.24	1.00	1.00	26.42	1.01
Typical quantity	14.22	2.01	12.05	2.94		
Largest quantity					62.42	12.49

N = 7,101 Linearized standard errors that account for the NESARC-III multistage sampling design are in parentheses. All models control for age, race/ethnicity, household income, and marital status.*p<.1; **p<.05; ***p<.01

*p<.1; **p<.05; ***p<.01

### Sensitivity analyses

Appendices B.1 through B.4 present results for the MCS and PCS regression results, including surface plots, along with the SF-6Dv1 results previously reported for direct comparison. All regressions control for age, race/ethnicity, household income, marital status, and confounding health conditions and are estimated separately for biological females and biological males. For biological females, the SF-6Dv1 and PCS results are very consistent, while the relationship between alcohol use behaviors and the MCS is relatively flat, suggesting that PCS plays a bigger role in the relationship between the frequency of consumption and health-related quality of life. For biological males, we see potentially offsetting effects with the MCS being concave in frequency and the PCS being convex in frequency. Both are mostly flat in quantity, though slightly downward sloping. Overall, the SF-6Dv1 results appear to be an approximate average of the MCS and PCS results, but the preference weighting may play an important role in this averaging. As a result, we cannot rule out the possibility that US-based preference weights would yield a slightly different relationship.

Appendices C.1 through C.4 present results stratified by age. As before, all regressions control for age, race/ethnicity, household income, marital status, and confounding health conditions and are estimated separately for biological females and biological males. Given the much smaller sample sizes, all results should be interpreted with caution. We see some changes in sign for individual parameters when compared to our main results, but these mostly reflect shifts in the functional form inflection points that have little bearing on the overall relationship over the logical range of the data. For biological females, results suggest a slightly more dynamic relationship between alcohol consumption patterns and health utility at younger ages. For older biological females, we see a relatively flat relationship, with a marked negative relationship between quantity and health utility for the oldest age group. For biological males, results may suggest a shift in the relationship between frequency of consumption and health utility from younger to older respondents. The estimated relationships may suggest a bimodal behavior pattern among young men such that health utility is greatest among either those who drink very infrequently or among those who drink daily. Among the older cohorts, the relationship flattens out considerably.

Appendices D.1 through D4 present results for the sample of individuals with alcohol use disorder. Our measures of the frequency and quantity of alcohol consumption are still strictly positive, and so our results are for the sample of individuals with alcohol use disorder who consumed alcohol in the past year. Those who abstained from alcohol in the past year are excluded. All regressions control for age, race/ethnicity, household income, and marital status, and are estimated separately for biological females and biological males. To better compare these results to those for the sample of people without alcohol use disorder, we present models both with and without controls for comorbid health conditions. For biological females, the relationship between alcohol consumption patterns and health utility is strikingly similar between the two samples, especially when comorbid health conditions are not controlled for. For biological males, we again see similar results for models that do not control for comorbid health conditions when compared to those for the sample of biological males without alcohol use disorder. When comorbid health conditions are controlled for, however, our results suggest that the relationship between health utility and the frequency of consumption is downward sloping for biological males with alcohol use disorder.

## Discussion

Low-risk drinking guidelines are a key part of many public health efforts to combat alcohol-related harms, yet evidence suggests that they are not effective in reducing alcohol consumption at a population level [13]. Moreover, the process for developing low-risk drinking guidelines has been criticized on a number of fronts, including as being paternalistic attempts to dictate the health risks that drinkers’ should accept [14, 15]. These concerns have led some to suggest alternative approaches to the development of guidelines [4, 5, 7, 14, 21]. Two basic approaches have emerged from these efforts: the relative risk approach [2] and the lifetime acceptable absolute risk approach [1].

The relative risk approach estimates mortality relative risk curves for alcohol consumption and sets drinking limits to minimize those risks. Guidelines based on relative risk, such as the 2011 Canadian guidelines [2], seek to find the pattern of alcohol use that maintains the relative risk of disease or death equal to or below that of abstinence. Guidelines that use absolute risk thresholds, such as the Australian [45] or current Canadian guidelines [10], define a level of absolute risk that is deemed “acceptable” and then seek to identify the amount or pattern of drinking that maintains absolute risk below this level. Regardless of the approach, guidelines’ primary focus on health morbidity and mortality means that individuals’ quality of life and personal priority for health are not explicitly considered when setting drinking limits.

The central premise of this analysis is that guidelines will be more effective in reducing population-wide levels of alcohol use if they incorporate quality of life and individuals’ preferences for health as additional considerations. Thus, we estimated the nonlinear relationship between health utility and the frequency and quantity of alcohol consumption. Health utility is a single measure that incorporates both quality of life and individuals’ preferences for health and so is ideal for our analysis. For females, health utility is concave in the frequency of alcohol consumption and decreasing in the quantity of consumption. For males, the overall relationship is flatter and possibly increasing in frequency though decreasing in quantity. Despite the differences, our results are broadly consistent with most low-risk drinking guidelines around the world: no more than 1 drink per day for women and no more than 2 drinks per day for men. Furthermore, our results support recommending that individuals not consume alcohol 7 days per week (i.e., we see no gains in health utility from daily drinking), particularly for females.

We find that the fundamental relationship between alcohol consumption patterns and health utility is largely stable for females, but sensitive to the model specification for males. In particular, the relationship among males appears to be influenced by the presence or absence of other health conditions that might impact health utility. In sensitivity analyses we explored differences across age groups and found potentially important differences in the relationship for biological females as they age that we did not observe for biological males, but the relatively small sample sizes for those results warrant caution in drawing definitive conclusions. Given the differences between biological females and males, our results suggest that efforts to develop a single set of drinking limits that applies to both men and women may be premature.

Beyond supporting the development of guidelines that are potentially more effective in public health efforts to reduce alcohol use in the general population, our results may have implications for alcohol use disorder prevention and treatment research as well. For example, alcohol screening and brief intervention is a common, non-abstinence-based intervention that encourages people to drink within low-risk guidelines [46, 47]. Furthermore, many policymakers and researchers are exploring non-abstinence targets for alcohol use disorder treatments such as pharmacotherapies [40, 41]. In sensitivity analyses that explored these relationships among individuals with alcohol use disorder, we found similar results for biological females when compared to our results for our main analysis sample. For biological males, we also found similar results when comorbid health conditions were not controlled for. These results may lend further support to non-abstinence targets for treatment efforts. Because heath utilities are the basis for quality-adjusted life years, treatment targets that explicitly incorporate health utilities may be both more acceptable to study subjects and improve treatment dissemination efforts by directly supporting cost-effectiveness analyses. Current research into alcohol harm reduction efforts [48–50] may also benefit from explicitly considering quality of life and individuals’ preferences as another area of potential harm caused by alcohol, as some research on alcohol’s harm to others has begun to do [51–53].

### Limitations

Although our results are generalizable to the US population and provide novel information that may inform low-risk drinking guidelines, they are descriptive and should not be interpreted as providing evidence of the causal link between alcohol consumption patterns and health utility. Furthermore, our analytic approach likely ascribed an undue level of precision to the alcohol consumption variables we used. The NESARC-III collected data on past year drinking and so recall errors likely reduce the precision of our consumption variables beyond the usual concern of under-reporting that is associated with self-reports of alcohol consumption.

Although less than 1% of the potential analysis sample had missing data on analysis variables, our use of listwise deletion is a potential limitation of our analysis. Some authors have noted that multiple imputation (MI) or full information maximum likelihood (FIML) are better approaches to dealing with missing alcohol use data in clinical trials of alcohol treatment [54]. These methods, however, may not be robust when the number of missing observations is relatively small [55, 56]. Furthermore, methodological guidance on missing data in secondary analyses of large survey datasets suggests that any bias created by listwise deletion is small when the proportion of observations with missing data is less than 10% [57]. In our analysis sample, only 0.09% of females (9 out of 9,897) and 0.1% of males (8 out of 7,109) had missing data. The very low percentage of missing data suggests that there is little gain to using more complicated missing data methods, and the low overall number of observations with missing data suggests that methods such as MI or FIML would likely not yield robust results.

Although we think incorporating health utility into low-risk drinking guideline development has the potential to greatly improve their usefulness, we also acknowledge that many other issues are relevant. People’s knowledge, or lack thereof, about standard drinks versus common serving sizes is likely a critical issue. Barriers to healthcare access limits the ability of healthcare providers to communicate the importance of guidelines and may also lead to excessive drinking as a form of coping. Perhaps most importantly, sociocultural and commercial influences on drinking behavior (e.g., perceived social norms and industry advertising) may well be the most salient determinant of drinking behavior for many people.

## Conclusion

We have shown that it is possible to incorporate health state preferences into the determination of drinking limits in a way that is consistent with the approaches used to assess the morbidity and mortality effects of alcohol. Moreover, our results suggest that incorporating preferences will not greatly change current recommendations, though more work is clearly needed to confirm this conclusion. Nonetheless, if we are correct in our motivating premise that guidelines will be more effective if they formally incorporate drinkers’ preferences for health, then our work serves as a critical starting point for a much broader research effort that has tremendous potential to reduce the harms associated with alcohol use by encouraging more people to adhere to low-risk drinking guidelines.

## Supplementary Information

Below is the link to the electronic supplementary material.

Supplementary Material 1

## Data Availability

This paper was prepared using a limited access dataset obtained from the National Institute on Alcohol Abuse and Alcoholism (NIAAA). The data are available upon request and with the permission of NIAAA. This paper has not been reviewed or endorsed by NIAAA and does not necessarily represent the opinions of NIAAA, who is not responsible for the contents.
