# Supplementary material for: Informing low-risk drinking guidelines: a secondary, cross-sectional data analysis of alcohol use and health utility
Source: Addict Sci Clin Pract. 2026 May 29;21:60. doi: 10.1186/s13722-026-00679-x (PMC13449395; doi:10.1186/s13722-026-00679-x)
Supplement: Supplementary file 1 [file 13722_2026_679_MOESM1_ESM.docx]

**Informing low-risk drinking guidelines: A secondary, cross-sectional data analysis of alcohol use and health utility**

Jeremy W. Bray^a*^

Arnie P. Aldridge^b^

Abraham Gebreselassie^a^

**Appendices**

# Appendix A: Prevalence of comorbid health conditions

|  | Biological females (N=9,888) | | Biological males (N=7,101) | |
| --- | --- | --- | --- | --- |
| Diagnosis | Proportion | Standard error^†^ | Proportion | Standard error^†^ |
| Human immunodeficiency virus | 0.005 | 0.001 | 0.005 | 0.001 |
| Cirrhosis | 0.002 | 0.000 | 0.002 | 0.000 |
| Other liver condition | 0.006 | 0.001 | 0.006 | 0.001 |
| Hardening of arteries | 0.008 | 0.001 | 0.016 | 0.002 |
| Diabetes | 0.071 | 0.003 | 0.086 | 0.004 |
| High blood pressure | 0.223 | 0.006 | 0.243 | 0.007 |
| High cholesterol | 0.180 | 0.005 | 0.195 | 0.006 |
| High triglycerides | 0.072 | 0.003 | 0.086 | 0.004 |
| Chest pain | 0.036 | 0.002 | 0.027 | 0.002 |
| Rapid heartbeat | 0.044 | 0.002 | 0.029 | 0.002 |
| Heart attack | 0.006 | 0.001 | 0.007 | 0.001 |
| Other heart condition | 0.041 | 0.002 | 0.042 | 0.003 |
| Stomach ulcer | 0.021 | 0.001 | 0.016 | 0.001 |
| Sexually transmitted disease | 0.008 | 0.001 | 0.004 | 0.001 |
| Epilepsy | 0.007 | 0.001 | 0.004 | 0.001 |
| Arthritis | 0.198 | 0.005 | 0.143 | 0.005 |
| Stroke | 0.006 | 0.001 | 0.007 | 0.001 |
| Difficulty sleeping | 0.081 | 0.004 | 0.047 | 0.003 |
| Liver cancer | 0.001 | 0.000 | 0.001 | 0.000 |
| Breast cancer | 0.010 | 0.001 | 0.000 | 0.000 |
| Cancer of the mouth, tongue, throat or esophagus | 0.002 | 0.000 | 0.001 | 0.000 |
| Other cancer | 0.028 | 0.002 | 0.034 | 0.003 |
| Anemia | 0.067 | 0.003 | 0.012 | 0.001 |
| Fibromyalgia | 0.032 | 0.002 | 0.003 | 0.001 |
| Nerve condition | 0.009 | 0.001 | 0.005 | 0.001 |
| Other nerve condition | 0.089 | 0.003 | 0.081 | 0.004 |
| Bowel condition | 0.043 | 0.003 | 0.017 | 0.002 |
| Osteoporosis | 0.058 | 0.003 | 0.009 | 0.001 |
| Lung condition | 0.048 | 0.002 | 0.039 | 0.003 |
| Pancreatitis | 0.002 | 0.001 | 0.003 | 0.001 |
| Tuberculosis | 0.001 | 0.000 | 0.001 | 0.000 |
| Traumatic brain injury | 0.004 | 0.001 | 0.004 | 0.001 |
| Depressive disorders | 0.245 | 0.007 | 0.121 | 0.004 |
| Bipolar disorder | 0.013 | 0.001 | 0.012 | 0.001 |
| Phobias | 0.077 | 0.003 | 0.037 | 0.002 |
| Anxiety disorders | 0.114 | 0.004 | 0.052 | 0.003 |
| Post-traumatic stress disorder | 0.043 | 0.003 | 0.017 | 0.002 |
| Schizotypal disorder | 0.041 | 0.002 | 0.044 | 0.003 |
| Eating disorders | 0.013 | 0.001 | 0.003 | 0.001 |
| Personality disorders | 0.085 | 0.003 | 0.089 | 0.004 |
| Nicotine use disorder | 0.144 | 0.004 | 0.175 | 0.005 |
| Cannabis use disorder | 0.007 | 0.001 | 0.016 | 0.002 |
| Sedative use disorder | 0.003 | 0.001 | 0.001 | 0.000 |
| Heroin and opioids use disorder | 0.005 | 0.001 | 0.004 | 0.001 |
| Cocaine and stimulants use disorder | 0.002 | 0.000 | 0.002 | 0.001 |
| Other drugs use disorder | 0.001 | 0.000 | 0.001 | 0.000 |

^†^Linearized standard errors that account for the NESARC-III multistage sampling design.

Appendix B.1: SF-6Dv1, MCS, and PCS regression results for biological females as a function of alternative frequency and quantity consumption measures

|  | Typical frequency and typical quantity | | | Typical frequency and typical quantity with bingeing controls | | | Typical frequency and largest quantity | | |
| --- | --- | --- | --- | --- | --- | --- | --- | --- | --- |
|  | SF-6Dv1 | MCS | PCS | SF-6Dv1 | MCS | PCS | SF-6Dv1 | MCS | PCS |
| Typical frequency | 0.987^***^ | 0.0640 | 1.037^***^ | 1.105^***^ | 0.126 | 1.067^***^ | 0.785^**^ | 0.143 | 0.789^***^ |
|  | (0.259) | (0.209) | (0.198) | (0.274) | (0.223) | (0.210) | (0.268) | (0.207) | (0.198) |
| Typical frequency^2^ | -0.113^**^ | 0.000216 | -0.108^***^ | -0.118^**^ | -0.00452 | -0.106^***^ | -0.104^*^ | -0.0114 | -0.0881^**^ |
|  | (0.0396) | (0.0299) | (0.0281) | (0.0401) | (0.0302) | (0.0287) | (0.0402) | (0.0300) | (0.0286) |
| Typical quantity | -0.190 | -0.364^**^ | 0.224^*^ | -0.141 | -0.314^*^ | 0.183 |  |  |  |
|  | (0.203) | (0.137) | (0.112) | (0.209) | (0.147) | (0.120) |  |  |  |
| Typical quantity^2^ | -0.0166 | -0.00136 | -0.0300^**^ | -0.0207 | -0.00356 | -0.0310^**^ |  |  |  |
|  | (0.0214) | (0.0111) | (0.0113) | (0.0208) | (0.0112) | (0.0114) |  |  |  |
| Typical frequency × typical quantity | -0.0520 | 0.0241 | -0.0791 | -0.109 | 0.00409 | -0.114^*^ |  |  |  |
|  | (0.0486) | (0.0405) | (0.0414) | (0.0603) | (0.0522) | (0.0499) |  |  |  |
| Largest quantity |  |  |  |  |  |  | -0.0510 | -0.239^***^ | 0.172^**^ |
|  |  |  |  |  |  |  | (0.0897) | (0.0690) | (0.0654) |
| Largest quantity^2^ |  |  |  |  |  |  | -0.00281 | 0.00315 | -0.00414 |
|  |  |  |  |  |  |  | (0.00291) | (0.00163) | (0.00333) |
| Typical frequency × largest quantity | | |  |  |  |  | 0.0108 | 0.0188 | -0.0188 |
|  |  |  |  |  |  |  | (0.0334) | (0.0241) | (0.0223) |
| **χ^2^ tests of joint significance** | | |  |  |  |  |  |  |  |
| Typical frequency and typical frequency^2^ | 7.90 | 0.21 | 15.11 | 8.79 | 0.38 | 14.20 | 4.25 | 0.33 | 8.28 |
| p value | 0.0006 | 0.8109 | 0.0000 | 0.0003 | 0.6838 | 0.0000 | 0.0166 | 0.7188 | 0.0004 |
| Typical quantity and typical quantity^2^ | 2.71 | 6.81 | 3.55 | 2.32 | 4.73 | 3.74 |  |  |  |
| p value | 0.0706 | 0.0016 | 0.0319 | 0.1029 | 0.0106 | 0.0268 |  |  |  |
| Largest quantity and largest quantity^2^ | | | |  |  |  | 2.08 | 6.87 | 3.73 |
| p value |  |  |  |  |  |  | 0.1296 | 0.0015 | 0.0269 |
| All drinking | 4.97 | 3.58 | 10.70 | 5.93 | 3.08 | 11.32 | 2.38 | 3.61 | 9.16 |
| p value | 0.0004 | 0.0049 | 0.0000 | 0.0001 | 0.0121 | 0.0000 | 0.0433 | 0.0047 | 0.0000 |
| **Inflection points** |  |  |  |  |  |  |  |  |  |
| Typical frequency | 8.88 | 14.81 | 6.64 | -29.13 | -8.04 | 235.87 | 3.65 | 10.837 | 2.99 |
| Typical quantity | -19.61 | -2.93 | -5.02 | 73.30 | -48.61 | -429.02 |  |  |  |
| Largest quantity |  |  |  |  |  |  | -2.06 | 5.56 | 13.94 |

N = 9,888

Linearized standard errors that account for the NESARC-III multistage sampling design are in parentheses. All models control for age, race/ethnicity, household income, marital status, and confounding health conditions.

^*^p<.1; ^**^p<.05; ^***^p<.01

Appendix B.2: Surface plots of regression results for biological females with health controls

| SF-6Dv1 health utility | MCS | PCS |
| --- | --- | --- |
| Typical frequency and typical quantity | | |
|  |  |  |
| Typical frequency and typical quantity controlling for bingeing | | |
|  |  |  |
| Typical frequency and largest quantity | | |
|  |  |  |

Appendix B.3: SF-6Dv1, MCS, and PCS regression results for biological males as a function of alternative frequency and quantity consumption measures

|  | Typical frequency and typical quantity | | | Typical frequency and typical quantity with bingeing controls | | | Typical frequency and largest quantity | | |
| --- | --- | --- | --- | --- | --- | --- | --- | --- | --- |
|  | SF-6Dv1 | MCS | PCS | SF-6Dv1 | MCS | PCS | SF-6Dv1 | MCS | PCS |
| Typical frequency | 0.114 | -0.376^*^ | 1.150^***^ | 0.162 | -0.358^*^ | 1.168^***^ | -0.177 | -0.556^***^ | 0.954^***^ |
|  | (0.273) | (0.161) | (0.206) | (0.279) | (0.165) | (0.202) | (0.285) | (0.152) | (0.203) |
| Typical frequency^2^ | 0.0419 | 0.0775^**^ | -0.119^***^ | 0.0377 | 0.0756^**^ | -0.118^***^ | 0.0529 | 0.0795^**^ | -0.108^***^ |
|  | (0.0426) | (0.0244) | (0.0302) | (0.0431) | (0.0244) | (0.0300) | (0.0441) | (0.0248) | (0.0308) |
| Typical quantity | 0.0124 | -0.0320 | 0.0229 | 0.0818 | 0.00156 | 0.0179 |  |  |  |
|  | (0.136) | (0.0858) | (0.0878) | (0.142) | (0.0917) | (0.0932) |  |  |  |
| Typical quantity^2^ | 0.000878 | -0.00165 | 0.00175 | -0.000158 | -0.00209 | 0.00200 |  |  |  |
|  | (0.00422) | (0.00298) | (0.00203) | (0.00417) | (0.00295) | (0.00213) |  |  |  |
| Typical frequency × typical quantity | -0.0669 | -0.0249 | -0.0805^**^ | -0.0809 | -0.0294 | -0.0951^***^ |  |  |  |
|  | (0.0396) | (0.0268) | (0.0267) | (0.0410) | (0.0281) | (0.0278) |  |  |  |
| Largest quantity |  |  |  |  |  |  | 0.0662 | -0.00436 | 0.0922 |
|  |  |  |  |  |  |  | (0.0971) | (0.0640) | (0.0538) |
| Largest quantity^2^ |  |  |  |  |  |  | -0.00288 | -0.00213 | -0.00322 |
|  |  |  |  |  |  |  | (0.00413) | (0.00249) | (0.00218) |
| Typical frequency × largest quantity | | |  |  |  |  | 0.00560 | 0.0197 | -0.0196 |
|  |  |  |  |  |  |  | (0.0203) | (0.0117) | (0.0128) |
| **χ^2^ tests of joint significance** | | |  |  |  |  |  |  |  |
| Typical frequency and typical frequency^2^ | 5.85 | 5.59 | 20.65 | 6.35 | 5.62 | 22.94 | 1.30 | 6.66 | 13.16 |
| p value | 0.0038 | 0.0049 | 0.0000 | 0.0024 | 0.0047 | 0.0000 | 0.2758 | 0.0019 | 0.0000 |
| Typical quantity and typical quantity^2^ | 0.08 | 0.62 | 3.45 | 0.33 | 0.43 | 3.94 |  |  |  |
| p value | 0.9195 | 0.5396 | 0.0352 | 0.7176 | 0.6495 | 0.0221 |  |  |  |
| Largest quantity and largest quantity^2^ | | | |  |  |  | 0.27 | 1.05 | 1.55 |
| p value |  |  |  |  |  |  | 0.7648 | 0.3536 | 0.2159 |
| All drinking | 3.07 | 2.96 | 9.59 | 2.93 | 2.66 | 10.69 | 1.80 | 3.02 | 8.26 |
| p value | 0.0124 | 0.0152 | 0.0000 | 0.0162 | 0.0261 | 0.0000 | 0.1192 | 0.0137 | 0.0000 |
| **Inflection points** |  |  |  |  |  |  |  |  |  |
| Typical frequency | 0.24 | 0.40 | 0.80 | 1.00 | 1.03 | 0.637 | 1.01 | 2.30 | 4.32 |
| Typical quantity | 2.01 | -12.64 | 11.91 | 2.94 | -6.87 | 10.70 |  |  |  |
| Largest quantity |  |  |  |  |  |  | 12.49 | 9.66 | 1.18 |

N = 7,101

Linearized standard errors that account for the NESARC-III multistage sampling design are in parentheses. All models control for age, race/ethnicity, household income, marital status, and confounding health conditions.

^*^p<.1; ^**^p<.05; ^***^p<.01

Appendix B.4: Surface plots of regression results for biological males with health controls

| SF-6Dv1 health utility | MCS | PCS |
| --- | --- | --- |
| Typical frequency and typical quantity | | |
|  |  |  |
| Typical frequency and typical quantity controlling for bingeing | | |
|  |  |  |
| Typical frequency and largest quantity | | |
|  |  |  |

Appendix C.1: Regression results for biological females as a function of alternative frequency and quantity of consumption measures by age groups

|  | Typical frequency and typical quantity | | | Typical frequency and typical quantity with bingeing controls | | | Typical frequency and largest quantity | | |
| --- | --- | --- | --- | --- | --- | --- | --- | --- | --- |
|  | 18-35 | 36-50 | 51+ | 18-35 | 36-50 | 51+ | 18-35 | 36-50 | 51+ |
| Typical frequency | -0.119 | 0.0174 | 0.0818 | 0.0523 | 0.163 | -0.0102 | 0.124 | 0.112 | 0.0576 |
|  | (0.335) | (0.358) | (0.257) | (0.377) | (0.405) | (0.264) | (0.362) | (0.359) | (0.258) |
| Typical frequency^2^ | -0.0260 | 0.0217 | 0.0132 | -0.0427 | 0.00834 | 0.0190 | -0.0505 | 0.00446 | 0.00922 |
|  | (0.0521) | (0.0542) | (0.0387) | (0.0542) | (0.0544) | (0.0381) | (0.0539) | (0.0536) | (0.0385) |
| Typical quantity | -0.480^*^ | -0.233 | -0.485 | -0.416 | -0.150 | -0.605 |  |  |  |
|  | (0.195) | (0.389) | (0.372) | (0.215) | (0.408) | (0.342) |  |  |  |
| Typical quantity^2^ | 0.00557 | -0.00923 | -0.0122 | 0.00189 | -0.00819 | 0.00233 |  |  |  |
|  | (0.0142) | (0.0540) | (0.0474) | (0.0144) | (0.0535) | (0.0410) |  |  |  |
| Typical frequency × typical quantity | 0.156^*^ | 0.0188 | -0.0347 | 0.126 | -0.0223 | 0.00683 |  |  |  |
|  | (0.0701) | (0.0879) | (0.0819) | (0.0921) | (0.121) | (0.0877) |  |  |  |
| Largest quantity |  |  |  |  |  |  | -0.294^**^ | -0.160 | -0.0484 |
|  |  |  |  |  |  |  | (0.0983) | (0.199) | (0.191) |
| Largest quantity^2^ |  |  |  |  |  |  | 0.00511^*^ | -0.00647 | -0.0247 |
|  |  |  |  |  |  |  | (0.00219) | (0.0187) | (0.0155) |
| Typical frequency × largest quantity | | |  |  |  |  | 0.0656 | 0.0222 | -0.00189 |
|  |  |  |  |  |  |  | (0.0469) | (0.0483) | (0.0446) |
| **χ^2^ tests of joint significance** | | |  |  |  |  |  |  |  |
| Typical frequency and typical frequency^2^ | 0.93 | 0.30 | 0.66 | 0.68 | 0.38 | 0.33 | 0.60 | 0.19 | 0.37 |
| p value | 0.3959 | 0.7378 | 0.52 | 0.5096 | 0.6873 | 0.72 | 0.5531 | 0.8274 | 0.69 |
| Typical quantity and typical quantity^2^ | 5.52 | 1.23 | 2.02 | 3.52 | 0.47 | 2.47 |  |  |  |
| p value | 0.0052 | 0.2976 | 0.14 | 0.0328 | 0.6271 | 0.09 |  |  |  |
| Largest quantity and largest quantity^2^ | | | |  |  |  | 4.71 | 2.35 | 2.85 |
| p value |  |  |  |  |  |  | 0.0109 | 0.1002 | 0.06 |
| All drinking | 2.25 | 1.19 | 2.20 | 1.46 | 0.99 | 1.83 | 2.04 | 1.54 | 2.21 |
| p value | 0.0546 | 0.3178 | 0.06 | 0.2095 | 0.4257 | 0.11 | 0.0791 | 0.1834 | 0.0582 |
| **Inflection points** |  |  |  |  |  |  |  |  |  |
| Typical frequency | 2.95 | 3.52 | -10.20 | 3.25 | -7.82 | -31.30 | 3.858 | 3.45 | -3.21 |
| Typical quantity | 1.750 | -9.06 | -5.38 | 1.79 | 1.48 | 175.60 |  |  |  |
| Largest quantity |  |  |  |  |  |  | 4.04 | -6.45 | -0.86 |

18-35 N = 3,372; 36-50 N = 2,827; 51+ N = 3,689

Linearized standard errors that account for the NESARC-III multistage sampling design are in parentheses. All models control for age, race/ethnicity, marital status, and confounding health conditions.

^*^p<.1; ^**^p<.05; ^***^p<.01

Appendix C.2: Surface plots of regression results for biological females with health controls by age groups

| 18-35 | 36-50 | 51+ |
| --- | --- | --- |
| Typical frequency and typical quantity | | |
|  |  |  |
| Typical frequency and typical quantity controlling for bingeing | | |
|  |  |  |
| Typical frequency and largest quantity | | |
|  |  |  |

Appendix C.3: Regression results for biological males as a function of alternative frequency and quantity of consumption measures by age groups

|  | Typical frequency and typical quantity | | | Typical frequency and typical quantity with bingeing controls | | | Typical frequency and largest quantity | | |
| --- | --- | --- | --- | --- | --- | --- | --- | --- | --- |
|  | 18-35 | 36-50 | 51+ | 18-35 | 36-50 | 51+ | 18-35 | 36-50 | 51+ |
| Typical frequency | -0.782^**^ | -0.287 | -0.273 | -0.665^*^ | -0.295 | -0.294 | -1.080^***^ | -0.587 | -0.229 |
|  | (0.273) | (0.299) | (0.224) | (0.287) | (0.311) | (0.223) | (0.294) | (0.305) | (0.222) |
| Typical frequency^2^ | 0.137^**^ | 0.0646 | 0.0616^*^ | 0.124^*^ | 0.0650 | 0.0640^*^ | 0.140^**^ | 0.0652 | 0.0382 |
|  | (0.0485) | (0.0465) | (0.0308) | (0.0499) | (0.0457) | (0.0302) | (0.0514) | (0.0463) | (0.0331) |
| Typical quantity | 0.0405 | -0.205 | 0.233 | 0.140 | -0.275 | 0.228 |  |  |  |
|  | (0.121) | (0.232) | (0.333) | (0.131) | (0.253) | (0.330) |  |  |  |
| Typical quantity^2^ | -0.00311 | 0.00415 | -0.0302 | -0.00482 | 0.00741 | -0.0306 |  |  |  |
|  | (0.00329) | (0.0176) | (0.0214) | (0.00334) | (0.0183) | (0.0214) |  |  |  |
| Typical frequency × typical quantity | -0.0567 | -0.00231 | -0.0108 | -0.0729 | -0.00221 | -0.0102 |  |  |  |
|  | (0.0423) | (0.0453) | (0.0574) | (0.0449) | (0.0531) | (0.0588) |  |  |  |
| Largest quantity |  |  |  |  |  |  | 0.0393 | 0.0539 | -0.162 |
|  |  |  |  |  |  |  | (0.0856) | (0.0932) | (0.128) |
| Largest quantity^2^ |  |  |  |  |  |  | -0.00331 | -0.00502 | 0.000421 |
|  |  |  |  |  |  |  | (0.00263) | (0.00542) | (0.00581) |
| Typical frequency × largest quantity | | |  |  |  |  | 0.0130 | 0.0394 | 0.0311 |
|  |  |  |  |  |  |  | (0.0275) | (0.0215) | (0.0254) |
| **χ^2^ tests of joint significance** | | |  |  |  |  |  |  |  |
| Typical frequency and typical frequency^2^ | 4.44 | 1.10 | 2.43 | 3.17 | 1.15 | 2.64 | 6.70 | 1.97 | 0.66 |
| p value | 0.0140 | 0.3380 | 0.09 | 0.0458 | 0.3214 | 0.08 | 0.0018 | 0.1437 | 0.52 |
| Typical quantity and typical quantity^2^ | 0.56 | 0.82 | 2.24 | 1.03 | 0.96 | 2.15 |  |  |  |
| p value | 0.5737 | 0.4410 | 0.11 | 0.3600 | 0.3874 | 0.12 |  |  |  |
| Largest quantity and largest quantity^2^ | | | |  |  |  | 1.11 | 0.44 | 1.21 |
| p value |  |  |  |  |  |  | 0.3319 | 0.6468 | 0.30 |
| All drinking | 3.55 | 1.08 | 2.30 | 2.63 | 1.16 | 2.18 | 3.61 | 1.72 | 1.30 |
| p value | 0.0052 | 0.3779 | 0.05 | 0.0275 | 0.3361 | 0.06 | 0.0047 | 0.1353 | 0.27 |
| **Inflection points** |  |  |  |  |  |  |  |  |  |
| Typical frequency | 1.46 | 2.67 | 2.52 | 2.16 | 2.59 | 2.56 | 3.276 | 1.32 | 5.38 |
| Typical quantity | -6.75 | 25.39 | 3.40 | -1.81 | 18.96 | 3.31 |  |  |  |
| Largest quantity |  |  |  |  |  |  | 12.36 | 10.54 | -5.87 |

18-35 N = 2,337; 36-50 N = 1,974; 51+ N = 2,790

Linearized standard errors that account for the NESARC-III multistage sampling design are in parentheses. All models control for age, race/ethnicity, marital status, and confounding health conditions.

^*^p<.1; ^**^p<.05; ^***^p<.01

AppendixC.4: Surface plots of regression results for biological males with health controls by age groups

| 18-35 | 36-50 | 51+ |
| --- | --- | --- |
| Typical frequency and typical quantity | | |
|  |  |  |
| Typical frequency and typical quantity controlling for bingeing | | |
|  |  |  |
| Typical frequency and largest quantity | | |
|  |  |  |

Appendix D.1: SF-6Dv1 regression results for biological females with AUD as a function of alternative frequency and quantity consumption measures

|  | Typical frequency and typical quantity | | Typical frequency and typical quantity with bingeing controls | | Typical frequency and largest quantity | |
| --- | --- | --- | --- | --- | --- | --- |
|  | Without health controls | With health controls | Without health controls | With health controls | Without health controls | With health controls |
| Typical frequency | 2.390^***^ | 0.595 | 2.532^***^ | 0.639 | 2.614^***^ | 0.671^*^ |
|  | (0.347) | (0.321) | (0.372) | (0.328) | (0.362) | (0.335) |
| Typical frequency^2^ | -0.304^***^ | -0.0682 | -0.317^***^ | -0.0719 | -0.308^***^ | -0.0705 |
|  | (0.0553) | (0.0489) | (0.0568) | (0.0494) | (0.0566) | (0.0504) |
| Typical quantity | -0.538^**^ | -0.242 | -0.339 | -0.121 |  |  |
|  | (0.179) | (0.140) | (0.195) | (0.155) |  |  |
| Typical quantity^2^ | 0.00876 | 0.00129 | 0.00200 | -0.00295 |  |  |
|  | (0.00682) | (0.00500) | (0.00619) | (0.00492) |  |  |
| Typical frequency × typical quantity | -0.0644 | -0.0469 | -0.0594 | -0.0398 |  |  |
|  | (0.0331) | (0.0252) | (0.0377) | (0.0285) |  |  |
| Largest quantity |  |  |  |  | -0.212^*^ | -0.0658 |
|  |  |  |  |  | (0.0887) | (0.0855) |
| Largest quantity^2^ |  |  |  |  | 0.0061^***^ | 0.00293 |
|  |  |  |  |  | (0.00146) | (0.00155) |
| Typical frequency × largest quantity | | |  |  | -0.0567^**^ | -0.0347 |
|  |  |  |  |  | (0.0196) | (0.0183) |
| **χ^2^ tests of joint significance** | |  |  |  |  |  |
| Typical frequency and typical frequency^2^ | 23.98 | 1.82 | 23.24 | 1.98 | 27.10 | 2.13 |
| p value | 0.0000 | 0.1674 | 0.0000 | 0.1423 | 0.0000 | 0.1235 |
| Typical quantity and typical quantity^2^ | 5.15 | 2.61 | 2.88 | 2.13 |  |  |
| p value | 0.0072 | 0.0783 | 0.0603 | 0.1234 |  |  |
| Largest quantity and largest quantity^2^ | | |  |  | 8.74 | 1.84 |
| p value |  |  |  |  | 0.0003 | 0.1637 |
| All drinking | 17.24 | 4.20 | 14.04 | 2.89 | 13.15 | 2.03 |
| p value | 0.0000 | 0.0016 | 0.0000 | 0.0171 | 0.0000 | 0.0799 |
| **Inflection points** |  |  |  |  |  |  |
| Typical frequency | 0.49 | -3.84 | -1.65 | -11.69 | 1.85 | 0.81 |
| Typical quantity | 32.51 | 23.87 | 60.15 | 58.29 |  |  |
| Largest quantity |  |  |  |  | 25.98 | 16.03 |

N = 3,880.

Linearized standard errors that account for the NESARC-III multistage sampling design are in parentheses. All models control for age, race/ethnicity, and marital status.

^*^p<.1; ^**^p<.05; ^***^p<.01

Appendix D.2: Surface plots of regression results for biological females with AUD

| Without health controls | With health controls |
| --- | --- |
| Typical frequency and typical quantity | |
|  |  |
| Typical frequency and typical quantity controlling for bingeing | |
|  |  |
| Typical frequency and largest quantity | |
|  |  |

Appendix D.3: SF-6Dv1 regression results for biological males with AUD as a function of alternative frequency and quantity of consumption measures

|  | Typical frequency and typical quantity | | Typical frequency and typical quantity with bingeing controls | | Typical frequency and largest quantity | |
| --- | --- | --- | --- | --- | --- | --- |
|  | Without health controls | With health controls | Without health controls | With health controls | Without health controls | With health controls |
| Typical frequency | 1.101^**^ | 0.0793 | 1.315^***^ | 0.305 | 1.037^**^ | -0.0152 |
|  | (0.335) | (0.319) | (0.354) | (0.333) | (0.335) | (0.316) |
| Typical frequency^2^ | -0.147^***^ | -0.0322 | -0.170^***^ | -0.0558 | -0.143^**^ | -0.0319 |
|  | (0.0427) | (0.0397) | (0.0446) | (0.0412) | (0.0438) | (0.0409) |
| Typical quantity | -0.0607 | -0.0305 | 0.0258 | 0.0545 |  |  |
|  | (0.0973) | (0.0870) | (0.101) | (0.0903) |  |  |
| Typical quantity^2^ | 0.00148 | 0.000284 | 0.000730 | 0.000011 |  |  |
|  | (0.00179) | (0.00233) | (0.00175) | (0.00224) |  |  |
| Typical frequency × typical quantity | -0.0293 | -0.00949 | -0.0375 | -0.0233 |  |  |
|  | (0.0249) | (0.0216) | (0.0249) | (0.0222) |  |  |
| Largest quantity |  |  |  |  | 0.0673 | 0.0376 |
|  |  |  |  |  | (0.0634) | (0.0562) |
| Largest quantity^2^ |  |  |  |  | -0.000992 | -0.000062 |
|  |  |  |  |  | (0.00088) | (0.00095) |
| Typical frequency × largest quantity | | |  |  | -0.0140 | -0.000513 |
|  |  |  |  |  | (0.0136) | (0.0104) |
| **χ^2^ tests of joint significance** | |  |  |  |  |  |
| Typical frequency and typical frequency^2^ | 5.95 | 1.27 | 7.34 | 1.41 | 5.38 | 1.90 |
| p value | 0.0035 | 0.2847 | 0.0010 | 0.2476 | 0.0059 | 0.1548 |
| Typical quantity and typical quantity^2^ | 0.50 | 0.06 | 0.13 | 0.20 |  |  |
| p value | 0.6100 | 0.9409 | 0.8812 | 0.8202 |  |  |
| Largest quantity and largest quantity^2^ | | |  |  | 0.80 | 0.34 |
| p value |  |  |  |  | 0.4507 | 0.7152 |
| All drinking | 4.08 | 1.33 | 5.21 | 1.50 | 4.53 | 1.73 |
| p value | 0.0020 | 0.2572 | 0.0003 | 0.1968 | 0.0009 | 0.1346 |
| **Inflection points** |  |  |  |  |  |  |
| Typical frequency | 0.85 | -1.93 | 1.52 | 2.34 | 3.01 | -2.78 |
| Typical quantity | 28.94 | 21.47 | 21.27 | 1.90 |  |  |
| Largest quantity |  |  |  |  | 12.61 | 316.76 |

N = 4,889

Linearized standard errors that account for the NESARC-III multistage sampling design are in parentheses. All models control for age, race/ethnicity, and marital status.

^*^p<.1; ^**^p<.05; ^***^p<.01

Appendix D.4: Surface plots of regression results for biological males with AUD

| Without health controls | With health controls |
| --- | --- |
| Typical frequency and typical quantity | |
|  |  |
| Typical frequency and typical quantity controlling for bingeing | |
|  |  |
| Typical frequency and largest quantity | |
|  |  |
